# Comet assay analysis of single–stranded DNA breaks in circulating leukocytes of glaucoma patients

**Published:** 2008-08-29

**Authors:** M Mozaffarieh, A Schoetzau, M Sauter, M Grieshaber, S Orgül, O Golubnitschaja, J Flammer

**Affiliations:** 1 University Eye Clinic, Basel, Switzerland; 2 Experimentelle Radiologie und Strahlenbiologie, Bonn, Germany

## Abstract

**Purpose:**

To investigate the amount of single-stranded DNA breaks in circulating leukocytes of primary open-angle glaucoma (POAG) patients.

**Methods:**

A comparative quantification of DNA breaks was performed in circulating leukocytes of POAG patients and healthy controls. The following groups of subjects were compared: (1) POAG patients having primary vascular dysregulation (PVD), (2) POAG patients without PVD, (3) healthy controls with PVD, and (4) healthy controls without PVD. The damage to DNA resulting in single-stranded breaks was assessed by means of the alkaline comet assay in which the damaged DNA migrates out of the nucleus forming a tail, which can be quantified using image analysis. Damage was quantified as the comet tail moment, which represents the extent of DNA damage in individual cells.

**Results:**

Leukocytes of POAG patients exerted a significantly higher amount of comet tails, which are indicative of DNA damage, in comparison to control leukocytes (p<0.001). DNA breaks occurred particularly in the subgroup of POAG patients with PVD in comparison to glaucoma patients without PVD (p=0.002). In the control group, there was no significant difference between controls with PVD and controls without PVD (p=0.86).

**Conclusions:**

POAG patients with PVD have a significantly higher rate of DNA breaks than both POAG patients without PVD and healthy controls with and without PVD.

## Introduction

Approximately 0.000165% of the DNA of the human’s genome is damaged at any given time [1]. DNA damage can occur as double-strand breaks, which result from two damages in opposite strands of the DNA helix, or as single–strand breaks, which result when only one of the two strands of a double helix has a defect [2]. The amount of DNA breaks depends on different factors such as cell type, the age of the cell, and the extracellular environment [3-5]. Fortunately, DNA damage can be repaired by various mechanisms [6]. In the physiologic state, generation of DNA breaks and subsequent DNA repair is more or less balanced. In other words, there is a "steady-state" [7]. However, if the damage induced is greater than the repair capacity, the amount of DNA breaks increases, and it may finally contribute to the development of disease.

In primary open-angle glaucoma (POAG), an increased number of DNA breaks has been described both in the trabecular meshwork [8,9] and and systemically in the circulating leukocytes [10]. The purpose of this investigation is to confirm increased DNA breaks in the circulating leukocytes of POAG patients and to test whether there is an association between primary vascular dysregulation (PVD) and DNA breaks.

## Methods

### Subjects

Patients with primary open-angle glaucoma (POAG) were recruited from the University Eye Clinic in Basel, Switzerland between January 2006 and September 2007. Healthy volunteers that were age and sex matched to POAG patients were recruited after a notification in the University Clinic informing potential volunteers of the opportunity to participate in a scientific research project. Ethical approval was obtained from the local medical ethics committee, and written informed consent was received from all subjects before entry into the study. The study was designed and conducted in accordance with the tenets of Declaration of Helsinki.

Patients with POAG had to meet the following inclusion criteria: (1) treated intraocular (IOP) less than 23 mmHg on multiple measurements, (2) progressive changes in either visual field in at least three successive perimetric tests, (3) optic nerve cupping, (4) open angles on gonioscopy, and (5) the absence of alternative causes of optic neuropathy (e.g., other types of glaucoma). Both POAG patients and healthy subjects with any of the following criteria were excluded: a history of other ocular or systemic disease (e.g., diabetes mellitus), smoking, drug or alcohol abuse, trauma, infection, or inflammation. PVD was defined as being present if it was detected in both the patient history as well as by nailfold capillaromicroscopy. PVD was defined as being absent if the patient history for PVD was negative and the results of nailfold capillaromicroscopy were negative. Cases in which patient history and nailfold capillaromicroscopy were contradictory were excluded from the study. The following groups of subjects were compared: (1) POAG patients having PVD, (2) POAG patients without PVD, (3) healthy controls with PVD, and (4) healthy controls without PVD. Demographic data of the different groups of subjects are given in Table 1.

**Table 1:** Demographic data of the study groups.

	**Control**		**Glaucoma**		**p value**
	PVD-	PVD+	PVD-	PVD+	
N	8	6	6	8	
Age	57 (14)	46 (17)	46 (14)	46 (14)	n.s. (*)
Mean IOP	13 (2.4)	15 (2.7)	13 (1.5)	12 (2.0)	n.s. (*)
Male	37.5%	50%	50%	37.5%	n.s. (**)
Female	62.5%	50%	50%	62.5%	n.s. (**)

Age and IOP are expressed as mean (SD). An asterisk indicates that the p value was obtained by one way ANOVA, and a double asterisk indicates that the p value was obtained by Fisher’s exact test. PVD+: with a primary vascular dysregulation; PVD-: without a primary vascular dysregulation.

### Isolation of leukocytes

Blood samples (20 ml) anti-coagulated with heparin were obtained by venipuncture from glaucoma patients and controls (PVD and non-PVD in both groups). The leukocytes were isolated using Ficoll-Histopaque gradients ((Histopaque 1077; Sigma-Aldrich, Switzerland)) as previously described [10]. The leukocyte bands were removed from the interface between plasma and the histopaque layers of each tube and collected into one 50 ml tube. The total volume was brought to 50 ml with cold Dulbecco’s Modified Eagle Medium (DMEM; Gibco, Invitrogen, Basel,

Switzerland). The cell suspension was washed three times with DMEM, and the total number of cells was determined. Cells were finally suspended in phosphate buffered saline (PBS) and aliquoted into Eppendorf tubes at 10^7^ cells/tube. After centrifugation, cell pellets were stored at −80 °C.

### Single cell gel electrophoresis (Comet assay)

**Figure 1:**
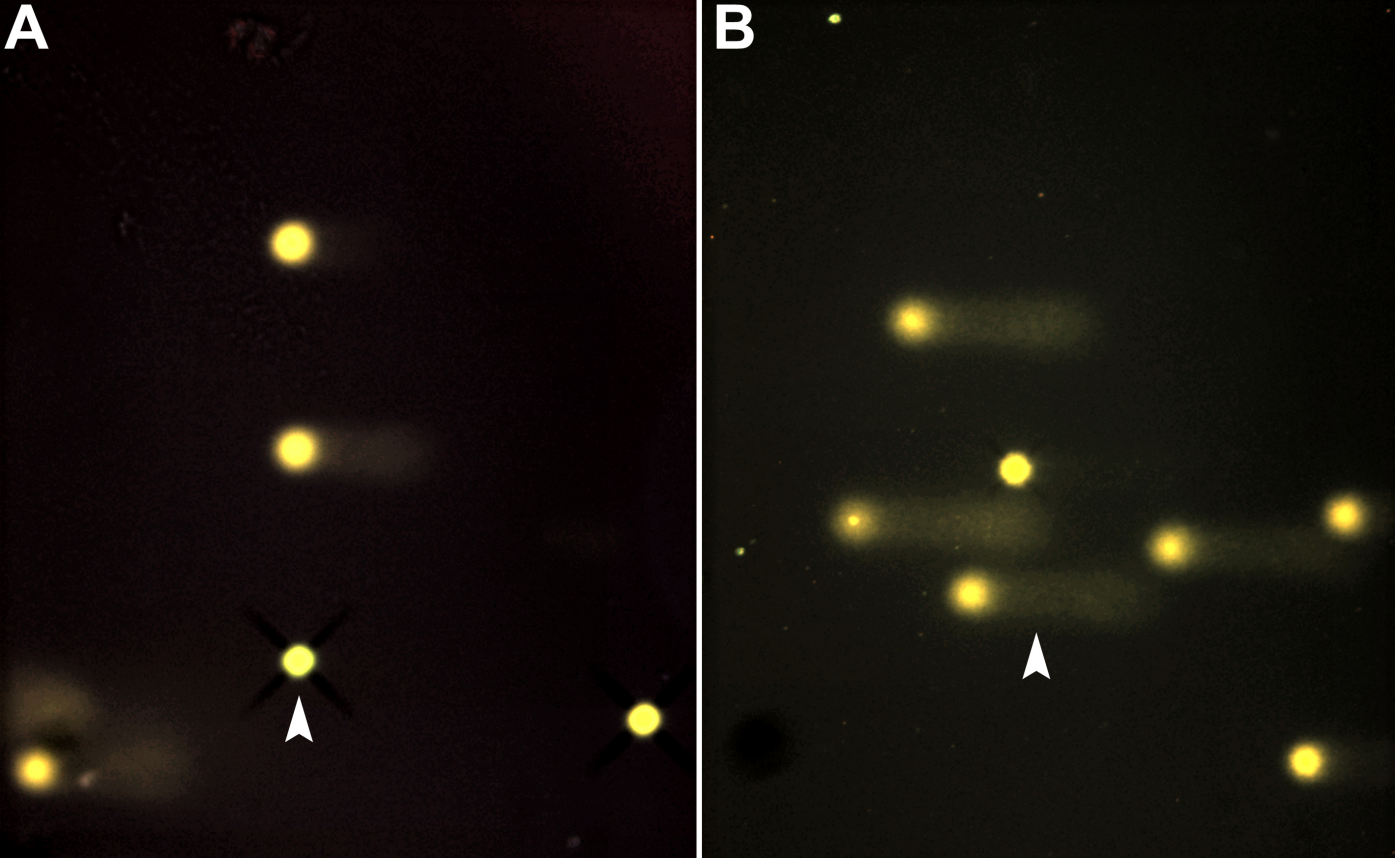
Photographs of cells analyzed by comet assay analysis. This figure shows photographs taken from the comet assay analysis. Each spot represents the DNA of an individual cell. The “dark/white” round spot represents the intact DNA. Intact DNA is a large molecule that does not migrate much in the electrophoretic field. The less dark “comet shaped” area adjacent to the nucleus represents DNA breaks that are small enough to move in the gel. The arrow in **A** points toward a virtually intact cell whereas the arrow in **B** points toward a cell with a large “comet”, which is indicative of a large amount of DNA breaks.

This simple, sensitive technique permits the detection of single-stranded DNA damage in single cells when performed in alkaline conditions. This method has previously been described in detail in the literature [11]. The cells under study are embedded in agarose on a slide and subjected to lysis followed by electrophoresis under specific conditions. During electrophoresis, the damaged and fragmented negatively charged DNA migrates away from the nucleus toward the anode. The amount of migrated DNA is a measure of the extent of DNA damage. To detect DNA, the slides are stained with cyber green and examined by fluorescence microscopy equipped with a personal computer based analysis system (Kinetic Imaging; Nikon, Zürich, Switzerland), which enables quantification of DNA damage. Cells containing damaged DNA have the appearance of a comet with a bright head and tail. In contrast, undamaged DNA appears as an intact nucleus with no tail (Figure 1).

### Quantification of DNA breaks

It is recommended by manufacturers that 50 cells on each slide be chosen at random for quantification of DNA damage using the computer software. The tail moment is defined as the product of the tail length and the fraction of total DNA in the tail (Tail moment=tail length  x % of DNA in the tail). This is calculated automatically by the computer software system as an average for the 50 cells selected for measurement.

In addition, a function known as the olive tail moment is automatically obtained by the computer software system for each cell analyzed. This parameter essentially represents the product of the percentage of total DNA in the tail and the distance between the centers of the mass of head and tail regions [Olive moment=(tail mean-head mean) x % of DNA in the tail]. All comets were quantified by three independent observers.

### Statistical analysis

The parameters used for the statistical evaluation were the tail moment and olive tail moment. As both parameters were zero-inflated (had many zeros), their distribution was heavy-tailed. The assumptions for usual regression modeling were therefore violated. To overcome this problem, the fraction of non-zero values compared to the total number of observations was counted for each subject. These fractions were approximately normally distributed. To detect the effect of the study group, PVD, and the observer on tail moment and olive moment, a linear mixed-effect model was performed. “Study group,” “observer,” and “PVD” are fixed effects of the model; “subject” was treated as a random effect. All possible interactions between the three main effects were included in the model.

The model also allowed for heteroscedasticity (unequal variances) in the factor levels. Additionally, the standard deviation between observers was calculated, treating “observer” as a random effect. All evaluations were performed using the statistical package R version 2.4.0 (SPSS 2006, Basel, Switzerland).

## Results

### Tail moment

Table 2 depicts the quantifications of DNA breaks (in the tail moment and olive moment) in controls and glaucoma patients. The data presents the mean, median, minimum, maximum, and standard deviation of fractions of non-zero values for each individual.

**Table 2:** Descriptive statistics for the tail moment and olive moment.

**Tail moment**						
	**Controls**			**Glaucomas**		
	**PVD-**	**PVD+**	**p value**	**PVD-**	**PVD+**	**p value**
Mean	0.38	0.36	0.86	0.45	0.72	0.002
Median	0.42	0.38		0.46	0.74	
StdDev	0.18	0.1		0.18	0.08	
Minimum	0.08	0.16		0.14	0.56	
Maximum	0.69	0.52		0.74	0.86	
N	24	18		18	24	
**Olive moment**						
	**Controls**			**Glaucomas**		
	**PVD-**	**PVD+**	**p value**	**PVD-**	**PVD+**	**p value**
Mean	0.49	0.53	0.6	0.61	0.78	0.02
Median	0.52	0.55		0.62	0.8	
StdDev	0.18	0.1		0.18	0.08	
Minimum	0.15	0.36		0.2	0.6	
Maximum	0.8	0.66		0.92	0.9	
N	24	18		18	24	

PVD+: with a primary vascular dysregulation; PVD-: without a primary vascular dysregulation.

**Figure 2:**
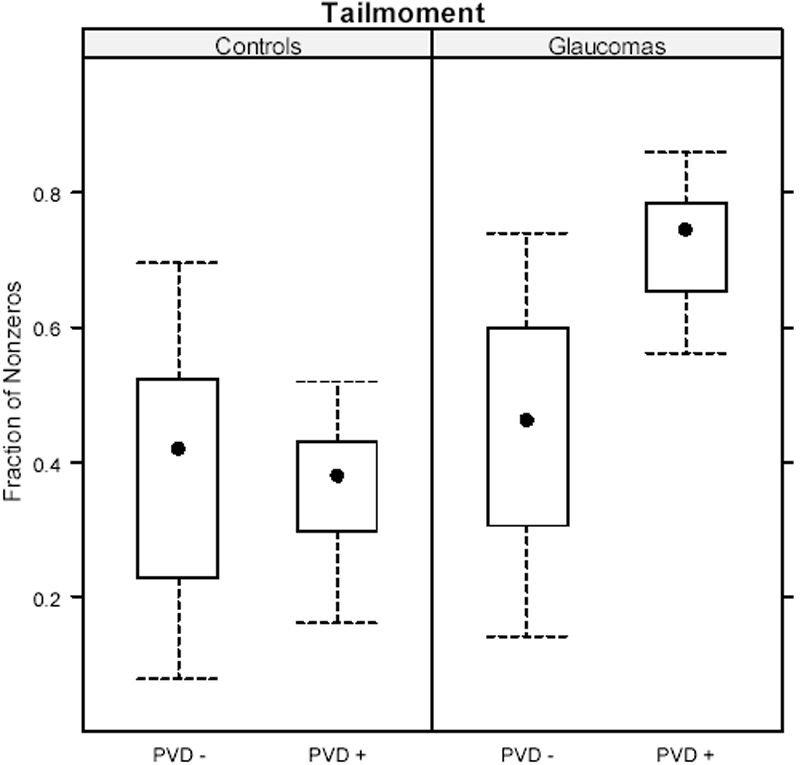
Comet assay analysis of the tail moment in glaucoma patients and controls. PVD+: with a primary vascular dysregulation; PVD-: without a primary vascular dysregulation.

POAG leukocytes exerted a significantly higher number of comet tails indicative of DNA damage in comparison to control leukocytes (p<0.001). There was a significant interaction between “PVD” and “study group” (p<0.001) as shown in Figure 2. This means that within the control group, there was no difference between the PVD and non-PVD subgroups (p=0.86). In the glaucoma group, however, there was a clear significant difference between PVD and non-PVD subgroups (p=0.002). The interactions of observers with other effects (study group and PVD) were not significant and were removed from the model. The inter-observer standard deviation was estimated as 12%.

### Olive moment

There were no significant interactions between any of the three factors. Therefore, the model was reduced to the three main effects. There were significant effects for “study group” (p<0.001) and “PVD” (p=0.046), and no significant effect for “observer” (p=0.12). The inter-observer standard deviation was estimated as 9%.

## Discussion

In this study we quantified single-stranded (ss) DNA breaks in circulating leukocytes of two subgroups of POAG patients, namely those with PVD and those without PVD. These results were compared to healthy subjects with and without PVD. Based on the results of the comet assay, we conclude that POAG patients with PVD have a significantly higher rate of DNA breaks than both POAG patients without PVD and healthy controls with and without PVD.

Single-stranded DNA breaks can result from a variety of factors including UV light [12], X-rays [13], ionizing radiation [14], toxins [15], chemicals [16], and reactive oxygen species (oxidative stress), all of which result in byproducts of normal metabolic processes [17]. The most likely reason for a higher rate of DNA breaks in POAG patients, especially in those patients having PVD, is increased oxidative stress. Oxidative stress occurs under a condition of high energy consumption, light exposure, or age-depending decline of coping capacity to deal with free radicals [18]. In glaucoma, an additional major factor is most likely a repeated mild reperfusion injury [19].

As a part of the systemic dysregulation, there is also some dysregulation of the ocular perfusion (disturbed autoregulation) [20,21]. As a consequence of disturbed autoregulation, fluctuation of IOP or blood pressure leads to a fluctuation of ocular perfusion and thereby to an unstable oxygen supply [22]. The resulting repeated mild reperfusion increases oxidative stress. Indeed, several findings indicate an increase in oxidative stress in glaucoma patients [8,9,23-28].

Among the controls, no significant differences were observed for single-stranded DNA breaks between the PVD and non-PVD groups. Taken together, our findings may indicate that PVD in glaucoma patients contributes to oxidative stress. It may, however, also indicate that PVD subjects have a less efficient antioxidant defense system. Preliminary studies show that glaucoma patients display a significant depletion of total antioxidant potential in their aqueous humor [24], a decrease in plasmatic glutathione levels [29], and an increase in serum antibodies against glutathione-S-transferase, which indicate reduced antioxidant defense in these patients [30]. However, the increase in DNA breaks in glaucoma patients with PVD may also reflect a weaker DNA repair capacity. Indeed, a different gene expression in the lymphocytes of glaucoma patients with PVD has been described both at the mRNA and at the protein level [31-34].

In summary, POAG patients with PVD have a significantly higher rate of DNA breaks than both POAG patients without PVD and healthy controls with and without PVD. Further investigations on the role of systemic antioxidant status and DNA repair capacity in these patients may have implications for understanding the pathophysiology of glaucoma.
